# Cystic squamous metaplasia in a giant benign phyllodes tumor of the breast: a case report

**DOI:** 10.1093/jscr/rjad402

**Published:** 2023-07-26

**Authors:** Evrard Niyonkuru, Simon Nisabwe, Zineb Sami, Issam Faizi, Asmae Mazti, Mohamed Elkarroumi, Mehdi Karkouri

**Affiliations:** Department of Pathology, Ibn Rochd University Hospital Center, Casablanca, Morocco; Faculty of Medicine and Pharmacy, Hassan II University of Casablanca, Casablanca, Morocco; Department of Pathology, Ibn Rochd University Hospital Center, Casablanca, Morocco; Faculty of Medicine and Pharmacy, Hassan II University of Casablanca, Casablanca, Morocco; Mohammed VI Center for Cancer Treatment, Ibn Rochd University Hospital Center, Casablanca, Morocco; Faculty of Medicine and Pharmacy, Hassan II University of Casablanca, Casablanca, Morocco; Mohammed VI Center for Cancer Treatment, Ibn Rochd University Hospital Center, Casablanca, Morocco; Faculty of Medicine and Pharmacy, Hassan II University of Casablanca, Casablanca, Morocco; Department of Pathology, Ibn Rochd University Hospital Center, Casablanca, Morocco; Faculty of Medicine and Pharmacy, Hassan II University of Casablanca, Casablanca, Morocco; Mohammed VI Center for Cancer Treatment, Ibn Rochd University Hospital Center, Casablanca, Morocco; Faculty of Medicine and Pharmacy, Hassan II University of Casablanca, Casablanca, Morocco; Department of Pathology, Ibn Rochd University Hospital Center, Casablanca, Morocco; Faculty of Medicine and Pharmacy, Hassan II University of Casablanca, Casablanca, Morocco

**Keywords:** benign phyllodes tumor, giant, squamous metaplasia, breast neoplasm, case report

## Abstract

Phyllodes tumor is a rare tumor of the breast, which encompasses both stromal and epithelial components. In these components, metaplastic changes can be observed occasionally. We report the case of a 51-year-old woman nulligest menopaused who presented a huge mass, largely ulcerated in her right breast. The radiological examination revealed a large tumor with microcalcifications classified as Breast Imaging and Reporting Data System Category 5. The patient undergone right mastectomy and the histological analysis revealed benign phyllodes tumor with cystic squamous metaplasia. Therefore, we aim to present this uncommon event occasionally occurring in phyllodes tumor of the breast.

## INTRODUCTION

Phyllodes tumor of the breast is a fibroepithelial neoplasm that shows prominent intracanalicular architectural pattern with leaf-like stromal fronds covered by double layer epithelial and accompanied by stromal hypercellularity. It is a rare form of breast tumors, accounting for 0.3–1% of all primary breast neoplasms [1]. Phyllodes tumors vary in size (i.e. can reach up to 60 cm) [2]. About 20% of all phyllodes tumors are larger than 10 cm in diameter and are considered as giant phyllodes tumors [1, 3]. Rarely, metaplastic changes can occur both in the epithelial and stromal component of those tumors. The stroma may show chondroid, osteoid or lipomatous metaplasia, whereas the epithelium occasionally shows apocrine or squamous metaplasia [4]. To the best of our knowledge, few cases of cystic squamous metaplasia in giant benign mammary phyllodes tumor have been reported in the literature. Therefore, the present work reports on the case of giant benign mammary phyllodes tumor with cystic squamous metaplasia. Moreover, it highlights the main details of this unusual case in the light of the literature.

## CASE REPORT

**Figure 1:**
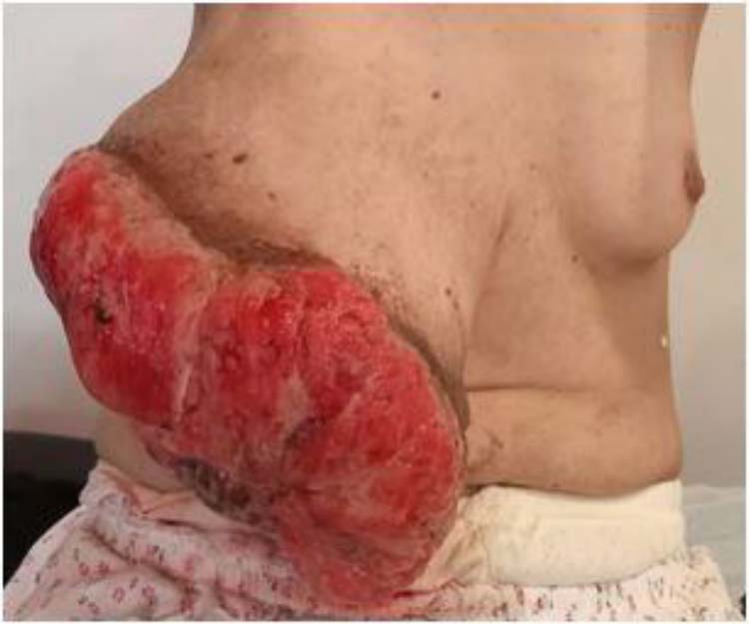
Clinical examination: mass ulcero-budding, occupying the whole right breast, with total destruction of the nipple-areolar plate.

A 51-year-old woman nulligest menopaused who presented a huge mass, largely ulcerated in her right breast. The patient had noticed a small and painless mass with no overlying skin changes 5 months earlier. She has undergone a biopsy suggesting benign phyllodes tumor. An excision of the mass had been proposed to her but she had refused it. The worsening of the symptoms pushed her to reconsult. Afterwards, the mass has increased considerably in size with skin manifestations progressing gradually to ulceration. There was no history of trauma. She had no specific medical history or family history of breast disease. Physical examination of right breast revealed a voluminous mass ulcero-budding that occupies the whole right breast and measures about 25 cm in size with total destruction of the nipple-areolar plate (Fig. 1). The contralateral breast examination was unremarkable. No axillary lymph nodes were palpable bilaterally.

**Figure 2:**
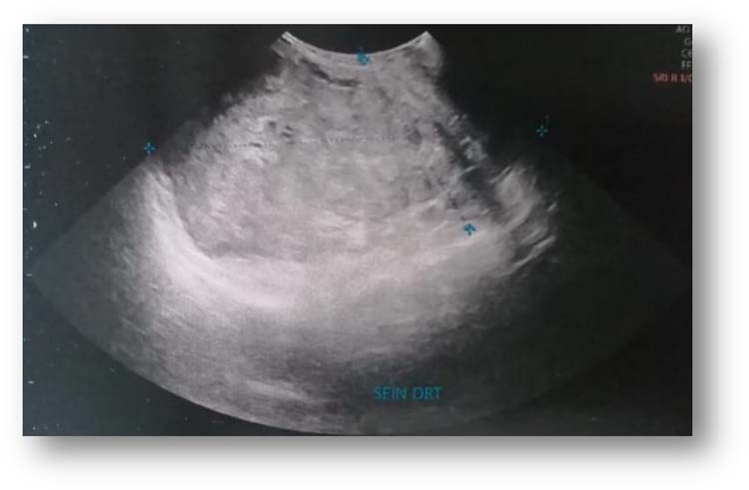
Ultrasonographic examination: large heterogenous hypoechoic mass with polylobed contours of 18 × 16 × 10 cm.

**Figure 3:**
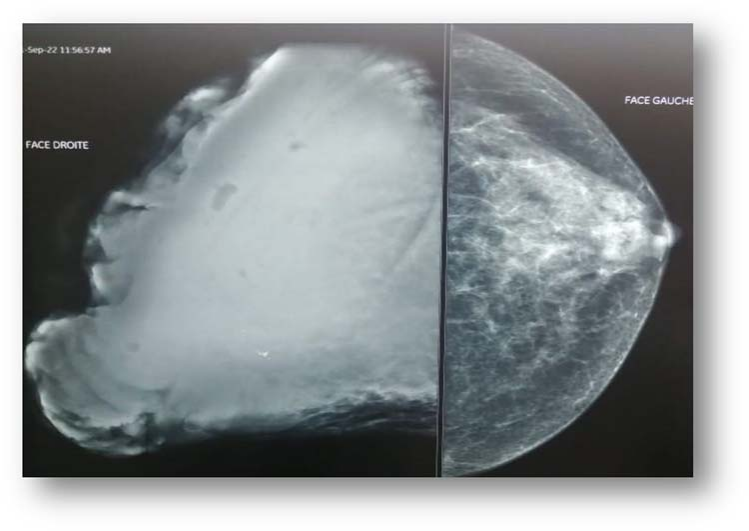
Mammography: voluminous opacity with a focus of vermicular microcalcifications.

Ultrasonographic examination showed large heterogenous hypoechoic mass vascular on doppler with polylobed contours of 18 × 16 × 10 cm (Fig. 2). Mammography showed an enlarged right breast through the presence of a voluminous opacity with a focus of vermicular microcalcifications (Fig. 3). The lesion was graded BI-RADS score 5 (Breast Imaging-Reporting and Data System score—American College of Radiology).

Concurrent computed tomography scan of the chest, abdomen and pelvis revealed a large right breast mass 22 × 16 × 16 cm ulcerated on its superficial face and infiltrating the pectoralis major muscle in depth enhanced after injection of the contrast product with significant vascularization from the internal mammary vessels. Moreover, there were no suspicious lesions at the pulmonary, abdomino-pelvic and bone level.

**Figure 4:**
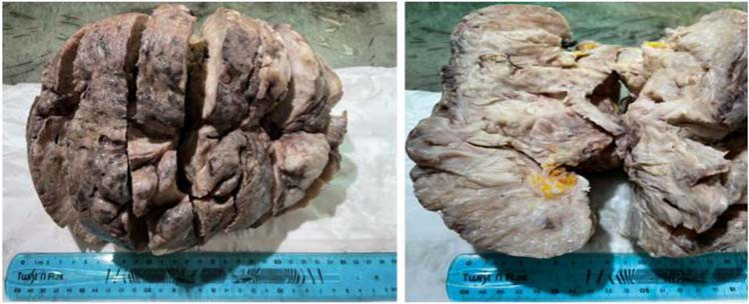
Macroscopic examination: mass, firm, focally fleshy appearance, myxoid and necrotic areas.

The patient has undergone right mastectomy with excision of deep margin in order to obtain tumor-free margins. The mastectomy specimen has been submitted to the histopathological examination. Macroscopic examination showed a mass 2398 g in weight and 27 × 18 × 14 cm in size, white beige, firm, focally fleshy appearance, myxoid and necrotic areas (Fig. 4).

**Figure 5:**
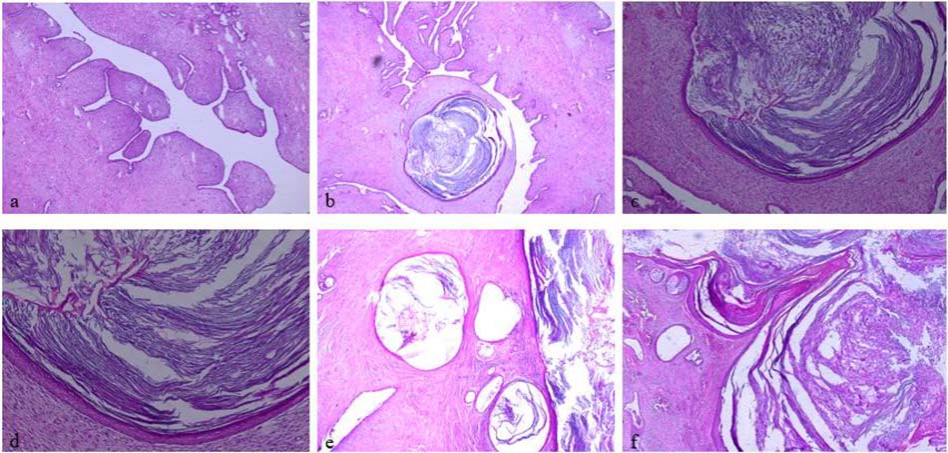
Histologic examination, hematoxylin and eosin stain: (**a**, ×5) showing typical leaf-like pattern with moderate cellularity of the stromal component. (**b**, ×5), (**e**, ×5) and (**f**, ×5) showing typical leaf-like pattern with moderate cellularity of the stromal component and cystic squamous metaplasia. (**c**, ×10) and (**d**, ×20) showing cystic squamous metaplasia.

The histological examination showed well limited fibro-epithelial tumoral proliferation. The mesenchymal component was moderately cellular and composed of spindle-shaped fibroblastic cells arranged in fascicles. The epithelial component was made often stretched or dilated and cystic glandular structures lined with a double cellular layer, epithelial-myoepithelial. No stromal overgrowth, cellular atypia or mitotic activity was observed. In addition, many variably sized cystic spaces lined by squamous epithelium were noted. These cystic spaces were filled with laminated keratin material (Fig. 5). The deep resection was tumor-free. According to the morphological findings, we diagnosed benign phyllodes tumor with cystic squamous metaplasia. The postoperative course was uneventful.

## DISCUSSION

Phyllodes tumor is a rare fibroepithelial neoplasm, but the occurrence of cystic squamous metaplasia is very uncommon. Tan et al. found epithelial squamous metaplasia in 12 cases from 335 reported cases, 5 (1.5%) of which revealed squamous metaplasia cysts [5]. Phyllodes tumor is usually diagnosed in women aged 31–49 years [6]. Clinically, it usually manifests as a mobile, unilateral, single, painless and rapid growing mass [6, 7]. The size of phyllodes tumors is quite variable but the median size is usually around 4 cm. The mass exceeding 10 cm is considered as a giant tumor [3]. The giant tumors distort the breast and may cause skin manifestations, such as an ulceration [7]. There is no significant relationship between phyllodes tumor size and histopathological sub-groups. Rapid growth may be detected in malignant tumors [8]. Radiologically, the imaging features of breast phyllodes tumor are nonspecific and appears as a well-circumscribed oval or lobulated mass with rounded borders [9]. It may be difficult to distinguish phyllodes tumor from cellular fibroadenoma, cysts, lipoma, hamartoma, sarcoma, well-circumscribed carcinoma and metastasis [7, 10]. Therefore, the histological examination remains the cornerstone of phyllodes tumor diagnosis and the establishment of histopathological subgroups.

Grossly, phyllodes tumor usually appears as lobulated tumor, with gray or red ‘meaty’ consistency, with hemorrhagic, fibro gelatinous, necrotic areas on cut section [11]. Phyllodes tumor is characterized by leaf-like phyllodes structures lined by double layered epithelium, an internal epithelium with a myoepithelium outside, that has cleft-like cystic spaces with hypercellular stroma and intracanalicular growth pattern [12].

Once the diagnosis of phyllodes tumor is made, it classified as benign, borderline or malignant according to the features, such as tumor margins, stromal overgrowth, tumor necrosis, cellular atypia and amount of mitosis per high-power field. The various changes can occur in the different components of phyllodes tumor. In the stromal component, pseudoangiomatous stromal hyperplasia, cartilaginous, osseous, lipomatous metaplasia or stromal giant cells can be seen. The epithelial component can undergo squamous or apocrine metaplasia but those changes are very uncommon [12]. In the present case, we have a squamous metaplasia arising in the benign phyllodes tumor.

The standard treatment for these tumors is surgical resection with tumor-free margins of 1 cm or larger to decrease the rate of recurrence. If the negative margins cannot be guaranteed through conservative surgery, simple mastectomy is recommended [11].

## CONCLUSION

Cystic squamous metaplasia is a rare feature of phyllodes tumor. Early detection of benign phyllodes tumor at small size allows breast conserving surgery.
